# Micro RNA-98 interferes with expression interleukin-10 in peripheral B cells of patients with lung cancer

**DOI:** 10.1038/srep32754

**Published:** 2016-09-08

**Authors:** Yun Li, Jian Rong, Jie Qin, Jin-yuan He, Hui-guo Chen, Shao-hong Huang

**Affiliations:** 1 Department of Cardiothoracic Surgery, the Third Affiliated Hospital, Sun Yat-sen University, Guangzhou, China; 2 Department of Anesthesiology, the First Affiliated Hospital, Sun Yat-sen University, Guangzhou, China; 3 Department of Radiology, the Third Affiliated Hospital, Sun Yat-sen University, Guangzhou, China

## Abstract

Interleukin (IL)-10-producing B cells (B10 cells) plays an important role in the tumor tolerance. High frequency of peripheral B10 cell was reported in patients with lung cancer recently. Micro RNA (miR) regulates some gene expression. This study test a hypothesis that miR-98 suppresses the expression of IL-10 in B cells of subjects with lung cancer. The results showed that the levels of miR-98 were significantly less in peripheral B cells of patients with lung cancer than that in healthy subjects. IL-10 mRNA levels in peripheral B cells were significantly higher in lung cancer patients as compared with healthy controls. A negative correlation was identified between miR-98 and IL-10 in peripheral B cells. Serum IL-13 was higher in lung cancer patients than that in healthy controls. The levels of IL-13 were also negatively correlated with IL-10 in B cells. Exposure B10 cells to IL-13 in the culture or over expression of miR-98 reduced the expression of IL-10 in B cells. Administration with miR-98-laden liposomes inhibited the lung cancer growth in a mouse model. In conclusion, up regulation of miR-98 inhibits the expression of IL-10 in B cells, which may contribute to inhibit the lung cancer tolerance in the body.

Lung cancer is one of the commonest cancers in the world and is one of the leading diseases of human death^1^. The pathogenesis of lung cancer is to be investigated^2^. The therapeutic remedies of lung cancer include surgery, chemotherapy, radiotherapy and some alternative remedies. The effects of lung cancer have been improved in recent years. However, the overall effects of lung cancer treatment are still poor^3^. Thus, to invent novel therapies for lung cancer is of significance.

Tumor tolerance is a phenomenon that the immune system of the body ignores the tumor growth in the body and allows tumors to grow out of control, in which tumors escape from the immune surveillance with unknown mechanisms^4^. The underlying mechanism of tumor tolerance is not fully understood yet^5^. Development of related tumor-tolerant immune cells is one of the reasons^6^. The immune tolerant cells include a number of cell types that produce immune tolerant molecules, such as IL-10, transforming growth factor (TGF)-beta, etc.^7^. The IL-10 producing B cells are one of the immune tolerant cell types^8^; these cells are also designated B10 cells. By producing IL-10, B10 cells are capable of suppressing other immune effector cell activities to compromise the anti-tumor ability of the body^9^. It is reported that the frequency of tumor tolerant immune cells increase in tumor-bearing subjects^10^. The mechanism remains to be further investigated.

Recent reports indicate that micro RNA (miR) is able to regulate the expression of some cytokines^11^. Mires are single stranded RNA chains with 18–22 nucleotides in length, which regulate target gene expression post-transcriptionally. Liu *et al*. reported that miR-98 inhibited the expression of IL-10 in macrophages^12^. Whether exogenous miR-98 can regulate the expression in B cells of tumor-bearing subjects has not been investigated. Based on the information above, we hypothesize that the impairment of miR-98 expression results in the over expression of IL-10 in B cells in patients with lung cancer. To test the hypothesis, we performed this study and found that the expression of miR-98 was significantly lower in peripheral B cells of patients with lung cancer as compared with healthy subjects. Administration with miR-98-carrying liposomes efficiently decreased the frequency of B10 cells in tumor-bearing mice and inhibited the experimental tumor growth.

## Materials and Methods

### Patients

Patients with non-small cell lung cancer were recruited into this study. The diagnosis of lung cancer was performed by our surgeons and pathologists. The demographic data of the patients are presented in Table 1. Patients with one of the following conditions were excluded: recurred lung cancer; using immune suppressive agents; with other severe organ diseases and with autoimmune diseases. The study procedures were approved by the Human Ethic Committee at Sun Yat-sen University. All the experiments were performed in accordance with the approved guidelines. An informed written consent was obtained from each human subject.

### Blood sample collection and mononuclear cell isolation

The peripheral blood samples (30 ml per person) were collected from each patient via ulnar vein puncture. The mononuclear cells (PBMC) were isolated from the blood samples by gradient density centrifugation.

### B cell isolation and culture

CD19 B cells were purified from the PBMCs by magnetic cell sorting with a commercial reagent kit (Mitenyi Biotech) following the manufacturer’s instructions. The purity of the B cells was greater than 98% as checked by flow cytometry. The B cells were cultured in RPMI1640 media supplemented with 10% fetal bovine serum, 100 U/ml penicillin, 0.1 mg/ml streptomycin, 2 mM L-glutamine and 20 ng/ml anti-CD40 mAb. The media were changed in 2 to 3 days. The viability of the cells was greater than 98% as assessed by Trypan blue assay before using for further experiments.

### Assessment of the frequency of peripheral B10 cells by flow cytometry

PBMCs were prepared as described above and stained with FITC-labeled anti-CD19 mAb or isotope IgG (BD Bioscience) for 30 min at 4 C, washed with phosphate-buffered saline (PBS) for 3 times, fixed with 1% paraformaldehyde for 30 min, incubated with 0.5% saponin for 10 min, stained with APC-labeled anti-IL-10 mAb (BD Bioscience) for 30 min at 4 C, washed with PBS for 3 times, and analyzed with a flow cytometer (BD Bioscience). The results were processed with software Flowjo (TreeStar). Data from the cells stained with isotope IgG were used as a gating reference.

### Determination of miR-98 and IL-10 mRNA in B cells by real time quantitative RT-PCR (RT-qPCR)

B cells were collected from the experiments. Total RNAs were extracted from the B cells with TRIzol reagent (Invitrogen). The first strand of DNA was synthesized with the RNA samples with a reverse transcription kit (Invitrogen) following the manufacturer’s instructions. The samples were then amplified in a real time PCR device (Bio rad) with the SYBR Green Master Mix and miR-98 primers (provided by the Enke Biotech, Shenzhen, China) and IL-10 primers (gttctttggggagccaacag and gctccctggtttctcttcct). The results were calculated with the 2^−ΔΔCt^ method and presented as fold change against controls.

### Preparation of miR-98 carrying liposomes

The miR-98 plasmids (Enke Biotech, Shenzhen, China) were constructed into liposomes following published procedures^13^. Briefly, 100 mg cholesterol, 100 mg 1,2-dioleoyl-sn-glycero-3-phospho-rac-(1-glycerol) sodium salt and 100 mg phosphatidylcholine were dissolved in 4.5 ml chloroform. The chloroform in the samples was evaporated to obtain a lipid film. 0.5 mg miR-98 plasmids or saline (control) in 0.5 ml water was added to 10 mg lipid film. After homogenizing for 10 min, the samples were heated and shaken for 15 min at 60 °C. The samples were then washed several times with distilled water.

### Over expression of miR-98 in B cells

The miR-98-carrying liposomes were added to B cell (isolated from healthy person blood samples) culture at 10 μg/ml. The cells were harvested 24 h later for further experiments. As analyzed by RT-qPCR, it resulted about 5 fold higher expression of the miR-98 in the B cells.

### Testing the effects of miR-98-carrying liposomes on suppression of IL-10 in B cells

B cells were isolated from the blood obtained from healthy persons. The miR-98 over expressing B cells and wild B cells were stimulated with LPS (1 μg/ml) for 48 h. The expression of IL-10 in the cells were analyzed by RT-qPCR.

### Development of a lung cancer-bearing mouse model

Male BALB/c mice were used to develop an experimental lung cancer-bearing mouse model. The mice were purchased from the Guangzhou Experimental Animal Center (Male, 6–8 week old). Lung cancer cell Line, KLN205 cells (ATCC, Beijing, China), was cultured in Dulbecco’s Modified Eagle Medium supplemented with 10% fetal bovine serum, 100 U/ml penicillin, 0.1 mg/ml streptomycin and 2 mM L-glutamine. The medium was changed daily. The cell viability was greater than 99% when used for further experiments as assessed by Trypn blue exclusion assay. The cells were injected into the subcutaneous of the side back skin at 10^6^ cells per mouse. The tumor size was measured and recorded daily. The using mouse in the study was approved by the Animal Ethic Committee at Sun Yat-sen University. The experiments were performed in accordance with the approved guidelines.

### Observation of the effects of miR-98-carrying liposome on inhibition of tumor growth

The tumor-bearing mice were intraperitoneally injected with miR-98-carrying liposomes (0.2 mg/mouse) or control liposomes on day 0, day 4 and day 8 respectively. The tumor size was recorded daily.

### Statistics

Data are normally distributed as analyzed by software Microsoft Excel Norm.DIST. The difference between two groups was determined by Student t test or ANOVA along with the Bonferroni correction if more than two groups. Correlation assay between two groups was performed with the software of Microsoft Excel. P < 0.05 was regarded as significant.

## Results

### High frequency of peripheral B10 cells in patients with lung cancer

**Figure 1:**
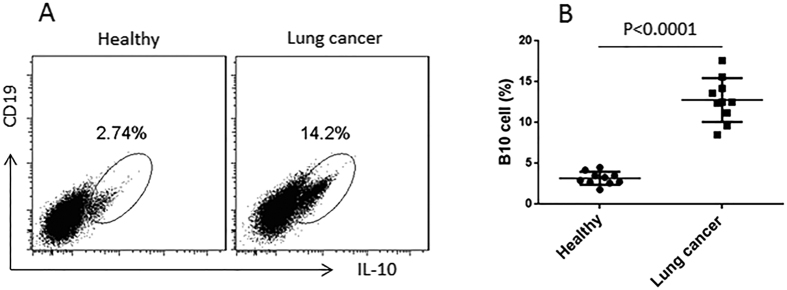
Peripheral B10 cells are more in patients with lung cancer. (**A**) the gated dot plots indicate the frequency of B10 cells in PBMCs of healthy subjects (n = 10) and patients with lung cancer (n = 10). (**B**) the dot plots show the individual value distribution of panel A.

B10 cells play a role in the tumor tolerance^14^. To understand the role of B10 cells in the pathogenesis of lung cancer, we collected the peripheral blood samples from NSCLC patients. PBMCs were isolated from the blood samples and analyzed by flow cytometry. The results showed that higher frequency of B10 cells (14.2%) was detected in the cancer group than that in the healthy control group (2.74%) (Fig. 1). The data demonstrate that B10 cells are increased in patients with lung cancer.

### Peripheral B cells express less miR-98 in patients with lung cancer

**Figure 2:**
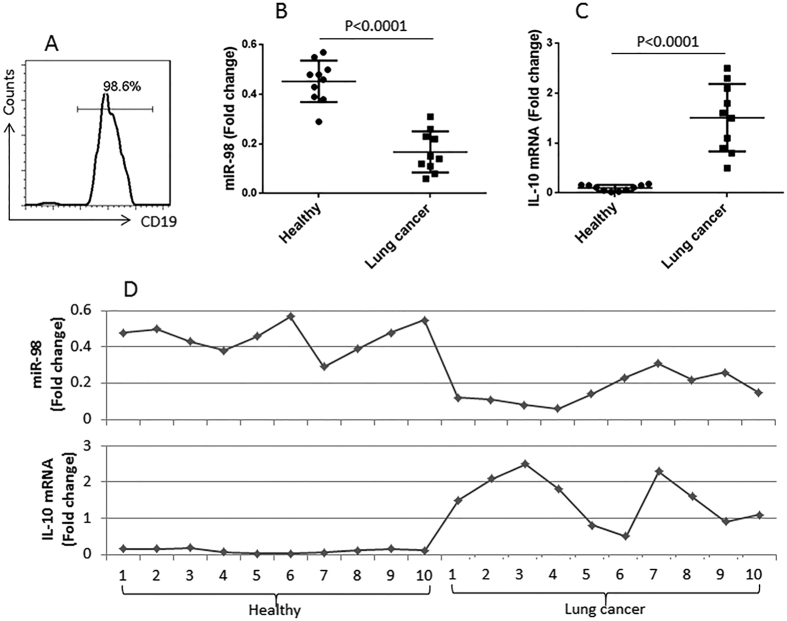
Assessment of miR-98 and IL-10 mRNA in peripheral B cells. (**A**) the histogram shows the purity of B cells isolated from PBMCs, which were isolated from the peripheral blood samples collected from healthy subjects (n = 10) and patients with lung cancer (n = 10). (**B,C**) the dispersed dot plots show the levels of miR-98 (**B**) and IL-10 mRNA in the isolated peripheral B cells. (**D**) the curves show the correlation of individual data between miR-98 and IL-10.

It is reported that miR-98 inhibits IL-10^12^. We wondered if miR-98 was inhibited in the B cells of patients with lung cancer. Next we isolated CD19^+^ B cells from PBMCs (Fig. 2A). The B cell extracts were analyzed by RT-qPCR for the expression of miR-98. The results showed that lower levels of miR-98 (0.19 folds vs controls) in peripheral B cells of lung cancer patients as compared with the healthy controls (0.44 folds vs controls) (Fig. 2B). Considering the expression of miR-98 might be associated with the expression of IL-10 in B cells, we measured the expression of IL-10 in peripheral B cells, which were significantly higher (1.55 folds vs 0.21 folds in the healthy group) in the cancer group (Fig. 2C). A correlation assay was performed with the data of miR-98 and IL-10 mRNA in B cells. The results showed a negative correlation (r = −0.7793, p < 0.0001) between miR-98 and IL-10 in peripheral B cells (Fig. 2D). The results implicate that miR-98 might be an inhibitor of IL-10 in B cells. The results implicate that the reduction of miR-98 expression in peripheral B cells may contribute to the increase in the expression of IL-10 in B cells.

### Over expression of miR-98 inhibits IL-10 in B cells

**Figure 3:**
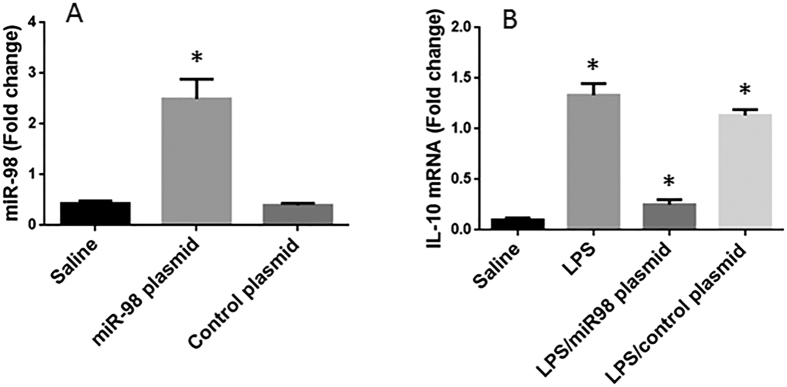
MiR-98 inhibits IL-10 expression in B cells. B cells were collected from healthy subjects. (**A**) the bars indicate the miR-98 levels in B cells after treatment with saline or miR-98 plasmids or control plasmids. (**B**) the bars indicate the IL-10 mRNA in the B cells after the treatment denoted on the X axis. Data of bars are presented as mean ± SD. *p < 0.01, compared with the saline group. The data were summarized from 3 independent experiments.

To elucidate the role of miR-98 in the regulation of IL-10 expression in B cells, we then prepared miR-98 over expressing B cells by transfecting B cells with miR-98 plasmids. The transfection increased about 6 folds higher expression of miR-98 in the B cells (Fig. 3A). The miR-98 over expressing B cells and wild B cells were stimulated with LPS to increase the expression of IL-10 in B cells. As analyzed by RT-qPCR, the wild B cells showed higher levels of IL -10 mRNA (1.46 folds vs controls) after exposure to LPS, while the miR-98 over expressing B cells had much lower levels of IL-10 mRNA as compared with the wild B cells (0.16 folds vs controls) (Fig. 3B). The results indicate that miR-98 does suppress the expression of IL-10 in B cells.

### MiR-98 liposomes inhibit lung cancer cell growth in a mouse model

**Figure 4:**
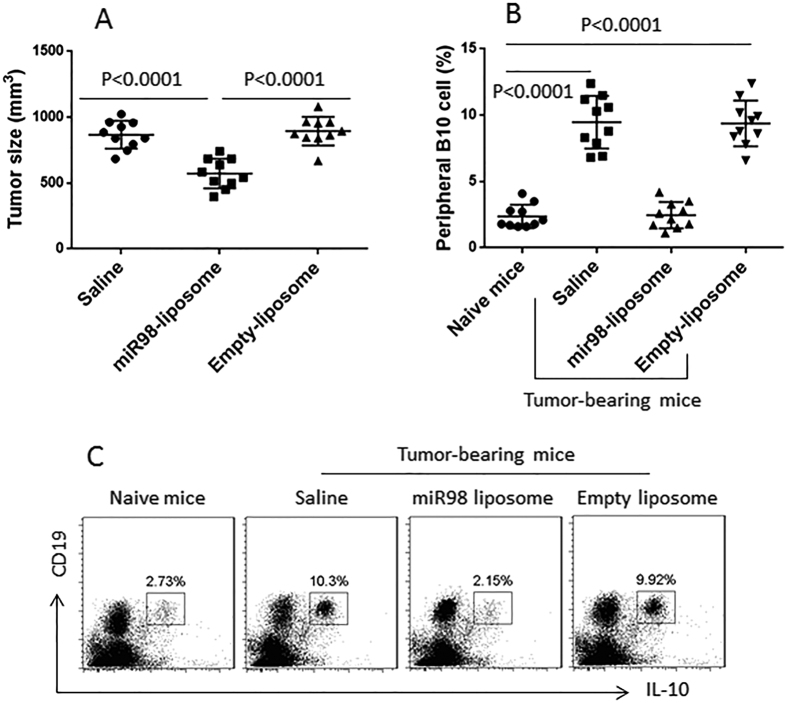
MiR-98-carrying liposomes inhibit B10 cells and suppress tumor growth. (**A**) the scatter plots show the tumor size in individual mice on day 12. (**B**) the scatter plots show the frequency of peripheral B10 cells. (**C**) the gated dot plots show the representative results of panel B. Each group consists of 10 mice.

Since B10 cells play an important role in the cancer-tolerance that contributes cancer cells to escape from the immune surveillance^8^, to inhibit IL-10 expression in B cells may inhibit cancer growth. To test this, we transplanted lung cancer cells to mice to induce a cancer mass. The cancer-bearing mice were intraperitoneal injection with miR-98 liposomes three times with 3 days apart from each injection. The cancer size was measured daily for 12 days. The results showed that the administration with miR-98 liposomes significantly inhibited the cancer growth (Fig. 4A). We also assessed the frequency of B10 cells in the peripheral blood that we collected at the sacrifice on day 12. The results showed that the frequency of B10 cells was significantly higher in cancer-bearing mice treated with saline than that of naive control mice. Administration with miR-98 liposomes significantly decreased the B10 cell frequency in cancer-bearing mice (Fig. 4B,C). The results demonstrate that administration with miR-98-laden liposomes can inhibit lung cancer growth in mice via suppression of IL-10 expression in B cells.

## Discussion

It is suggested that B10 cells contribute to tumor tolerance and tumor escaping from immune surveillence in the body^9^. The present data show that the frequency of B10 cells was higher in lung cancer patients than healthy controls. We asked if this phenomenon was a coincidence or it had a pathogenic significance in lung cancer. Further data showed supportive evidence that the peripheral B cells had lower levels of miR-98 and higher levels of IL-10 as compared with healthy controls; the two parameters had a negative correlation. This implicates that miR-98 can suppress the expression of IL-10 in B cells. The inference was supported by the data that administration with miR-98-carrying liposomes efficiently inhibited experimental tumor growth and down regulated the frequency of peripheral B10 cells in tumor-bearing mice. Although the miR-98-liposomes did not completely remove the tumor, it significantly reduced the tumor size, indicating this can be a supportive remedy to treat lung cancer.

In this study, we focused on observing the frequency of B10 cells in lung cancer patients. We found that the frequency of the peripheral B10 cells was significantly higher in cancer patients than that in healthy controls. The IL-10-producing T cells and B cells have immune regulatory functions, such as the type 1 regulatory T cells and B10 cells^15^,^16^. Upon activation by proper stimuli, the immune regulatory T cells or B cells release IL-10 to inhibit immune responses by suppressing other immune effector cell functions^17^. On one hand, it can be a beneficial reaction to the hosts; such as it can inhibit inflammation^17^. On the other hand, it can be a hazardous reaction in cancer patients since the immune regulatory cells can suppress tumor specific immunity, such as inhibit tumor specific CD8^+^ T cells^18^,^19^.

To find the mechanism by which IL-10 over expression in B cells of patients with cancer is of significance. Based on published data that miR-98 can inhibit IL-10 expression in macrophages^12^, the phenomenon of high frequency of B10 cells in cancer patients implicates that the miR-98 expression in B cells of lung cancer patients may be dysfunctional. The inference is supported by the data that the expression of miR-98 in B cells of lung cancer patients is markedly less than that in healthy controls. Others indicate that miR-98 can bind to the 3′UTR of the IL-10 gene to inhibit IL-10 expression because that IL-10 3′-UTR contains the miR-98 binding site^12^.

Employing liposomes as a carrier, we constructed the miR-98-carrying liposomes. After treating tumor-bearing mice with the miR-98-carrying liposomes, the tumor size and the frequency of peripheral B10 cells were significantly less as compared with that in control mice; the latter was treated with saline.

In summary, the present data reveal that the peripheral B cells of lung cancer patients express low levels of miR-98 and high levels of IL-10. To increase the expression of miR-98 inhibits the expression of IL-10 in B cells. The results suggest that such a miR-98-carrying liposomes can be a supportive therapy in the treatment of lung cancer.

## Additional Information

**How to cite this article**: Li, Y. *et al*. Micro RNA-98 interferes with expression interleukin-10 in peripheral B cells of patients with lung cancer. *Sci. Rep*. **6**, 32754; doi: 10.1038/srep32754 (2016).

## Figures and Tables

**Table 1:** Demographic data of patients with lung cancer.

Items	
**Male**	5 (50%)
**Female**	5 (50%)
**Age**	55.4 ± 15.6 years
**Cancer type**	NSCLC
**Smoking**	3 (30%)
**Recurrence**	0

NSCLC: Non-small cell lung cancer.
